# Adenomyosis: Back to the future?

**Published:** 2017-03

**Authors:** Z Ferraz, N Nogueira-Martins, F Nogueira-Martins

**Affiliations:** Obstetrics and Gynecology Unit, Coimbra University Hospital Centre, Coimbra, 3030-165, Portugal; Serviço de Ginecologia e Obstetrícia, Centro Hospitalar Tondela-Viseu EPE, Viseu, Av. Rei D. Duarte, 3504-509, Portugal.

**Keywords:** Adenomyosis, diagnosis, management, myometrium, symptoms

## Abstract

Adenomyosis is a benign invasion of endometrium into the myometrium. It produces a diffusely enlarged uterus and predominantly affects women in the late reproductive age. It has recently been associated with a negative impact on female fertility. Diagnosis is challenging because nonspecific symptoms can be present. However, it is asymptomatic in the majority of women. Medical treatment with GnRH agonist seems to be promising, particularly in subfertile women.

A retrospective study was performed (182 patients) by examination of histopathological results with “adenomyosis” between January/2013 and September/2016. The patients ranged in age from 34 to 78 years and the majority (94.5%) were over 40 years old. Most (90.6%) were multiparous. All women underwent a sonographic evaluation. More than a half had a heterogeneous myometrium (75.7%) and concomitant presence of myoma (65.4%). The majority of women were asymptomatic, nevertheless, when symptoms were present, menorrhagia and metrorrhagia were the most important complaints. About one third of women had had prior uterine surgery, 67.6% had had previous caesarean section(s). Medical treatment with progestatives was used by 61.3% of women and 23% tried more than one type of hormone therapy. Endometrial cancer was present in 5.5% of cases, all of which were lowgrade endometrioid adenocarcinoma.

Adenomyosis is an important challenge in gynaecology and often remains a post-operative diagnosis after hysterectomy. Clinicians should be on the watch for ultrasound features suggesting its presence to allow an early diagnosis. Future investments in the creation of diagnostic algorithms and alternative treatments are warranted.

## Introduction

Adenomyosis is a benign gynaecological disease that predominantly affects women in late reproductive age. The prevalence ranges from 5 to 70%; this high variability is due to several factors such as the diagnostic criteria, the characteristics of the sample under analysis, and the researcher’s skills (Graziano et al., 2015).

Adenomyosis was first described in 1860 by Carl von Rokitansky, who found endometrial glands in the myometrium and subsequently referred to this finding as “cystosarcoma adenoids uterinum”. The modern definition of adenomyosis was provided in 1972 by Bird who stated that adenomyosis may be defined as the “benign invasion of endometrium into the myometrium, producing a diffusely enlarged uterus which microscopically exhibits ectopic, non-neoplastic, endometrial glands and stroma surrounded by the hypertrophic and hyperplastic myometrium”. The presence of this ectopic endometrial tissue may be associated with weakness of the myometrium caused by trauma, such as caesarean section, dilatation and curettage, as well as myomectomy (Sun et al., 2010; Taran et al., 2013). Recently, it has been reported that adenomyosis is associated with endometrial carcinoma (Verit and Yucel, 2013).

Nonspecific symptoms can be present (dysmenorrhea, dyspareunia, chronic pelvic pain, abnormal vaginal bleeding and infertility), while a third of the women are asymptomatic (Graziano et al., 2015; Sun et al., 2010). Age up to 40-50 years, multiparity, prior uterine surgery, depression and anti-depressant use, and tamoxifen treatment are risk factors that predispose to adenomyosis.

At first, adenomyosis was diagnosed from histological specimens. Nowadays the improvement of diagnostic approaches allows the physicians to identify the disease by means of non-invasive and equally reliable instruments, such as sonography (transvaginal or abdominal) and magnetic resonance imaging (MRI) (Sun et al., 2010; Graziano et al., 2015). Minimally invasive techniques (sonohysterosalpingography, hysteroscopy and laparoscopy) can also be of use.

In clinical practice, homogeneous reporting of ultrasound findings of the myometrium is essential to reduce intra- and inter-observer variability. The Morphological Uterus Sonographic Assessment (MUSA) is a consensus report based on terms, definitions and measurements used to describe the sonographic features of the myometrium, describing the two most common myometrial lesions (fibroids and adenomyosis) and uterine smooth muscle tumours (Table I) (Van den Bosch et al., 2015). This consensus is likely to improve adenomyosis diagnosis. The terms and definitions used can help to produce a structured report when describing the sonographic appearance of the myometrium and myometrial lesions, and to harmonize the terminology. Ultrasound evaluation is a first-stage imaging technique for assessing the myometrium and several sonographic features have been reported for adenomyosis (Sun et al., 2010; Van den Bosch et al., 2015): (A) heterogeneous myometrium; (B) myometrial cysts; (C) sub-endometrial echogenic linear striations; (D) globular uterine enlargement (≥12 cm in uterine length, not explained by the presence of a myoma); (E) myometrial anteroposterior asymmetry (posterior wall is thicker than anterior wall); (F) poor identification of the endometrial junction; (G) thickening of the transition zone (this zone is a layer that appears as a hypoechoic halo surrounding the endometrial layer. A thickness of 12 mm or greater has also been shown to be associated with adenomyosis).

**Table I:** — Important features in diagnosis of myoma and adenomyosis FIGO (International Federation of Gynaecology and Obstetrics).

**Feature**	**Typical myoma**	**Adenomyosis**
Serosal contour of uterus	Lobulated or regular	Globally enlarged uterus
Definition of lesion	Well-defined	Ill-defined in diffuse adenomyosis (May be well-defined in adenomyoma)
Symmetry of uterine wall	Asymmetrical in presence of well-defined lesion	Myometrial anteroposterior asymmetry
Lesion	Well-defined outline Round/oval/lobulated Smooth contour Hypo/hyperechogenic rim Edge/internal shadow Uniform (hypo or hyperechogenic) Non-uniform (mixed echogenicity) Circumferential flow	Ill-defined outline Ill-defined shape Irregular/ill-defined contour No rim No edge shadow, fan-shaped shadowing Non-uniform (mixed echogenicity) Cysts, hyperechogenic islands Subendometrial lines and buds Translesional flow
Junctional zone (JZ)	JZ not thickened (regular or not visible) Interrupted JZ in areas with lesions types (1-3)	Thickened (irregular or ill-defined) Interrupted JZ (even in absence of localized lesions)

A prospective study was performed involving 72 premenopausal patients scheduled for hysterectomy for benign pathology (Exacoustos et al., 2011). They concluded that the presence of myometrial cysts was the most specific and heterogeneous myometrium was the most sensitive sign. Recently, a new technique using in vitro 3D ultrasound examination, with needle stereotaxis after hysterectomy, demonstrated that the ultrasound findings were compared with the macroscopical and the microscopical examination (Van den Bosch et al., 2016). This new approach can help in indicating the precise location of the preoperatively identified adenomyosis and may optimize the diagnostic accuracy of the histological examination in women with adenomyosis.

Adenomyosis is recurrently associated with a negative impact on women’s fertility (Wang et al., 2009; Tsui et al., 2015). However, the management of these women is highly controversial, so there is no consensus relative to conservative surgery. This means that treatment can be medical or surgical and the choice depends on whether the patient wishes to preserve fertility.

Medical treatments follow the principles of the management of endometriosis. Gonadotropinreleasing hormone (GnRH) agonist is probably the most popular and best accepted therapy (Wang et al., 2009; Tsui et al., 2015). This treatment aims the inhibition of ovulation, abolition of menstruation and achievement of a stable steroid hormone environment, based on the concept that the responses of the eutopic and ectopic endometrium are substantially similar (Tsui et al., 2015). Subfertile women should try GnRH agonist treatment first and afterwards undergo an active therapy strategy, such as conservative surgery, to improve fecundity (Wang et al., 2009). Recently a retrospective study was published involving four adolescents with adenomyosis diagnosed on pelvic MRI (Mansouri et al., 2015). All improved symptomatically after therapy with a GnRH agonist and follow-up, and MRI showed resolution of adenomyosis after three years. However, medical treatments are symptomatic and not cytoreductive; lesions survive the use of any drug, at any dose, for any length of time, and are ready to resume their metabolic activity once treatment is discontinued. Medical treatments are therefore associated with adverse effects and events, with an impact on longterm use and adherence (Wang et al., 2009).

The decision to use conservative surgery in the management of women with adenomyosis should be taken carefully, because conservative surgery can result in adhesion, distortion of the uterus, occlusion of the Fallopian tubes and the risk of total and/or subtotal hysterectomy, similar to other uterine surgical procedures (Tsui et al., 2015). Reproductive performance after conservative surgery seemed to be improved compared with that after GnRH agonist treatment.

Hysterectomy is the most effective treatment when fertility preservation is not desired. 

## Materials and Methods

A retrospective study was carried out based on the collection of histopathologic results, which included the diagnosis of “adenomyosis” (after hysterectomy or surgical hysteroscopy), between January 2013 and September 2016. The authors achieved a sample of 182 patients. The following clinical data was checked for each patient: age at diagnosis, parity, previous surgery, symptoms, medical treatments and sonographic findings. Statistical analysis was performed using SPSS 23.0.

## Results

In this retrospective study we included a total of 182 patients with diagnosis of adenomyosis based on histopathological results of hysterectomy specimens (94%; Table I) and biopsy performed by hysteroscopy. The patients ranged in age from 34 to 78 years (mean, 51.7±8.7 years). The majority of women (94.5%) were aged over 40. Most (90.6%) were multiparous. Infertility was mentioned by two women.

The main indications for hysterectomy were dysmenorrhea, abnormal vaginal bleeding, genital prolapse and chronic pelvic pain. In the hysteroscopy subgroup, the main indications were abnormal vaginal bleeding and abnormalities in the transvaginal ultrasound scan.

All women had undergone a sonographic evaluation (at our institution or elsewhere). The majority of patients (75.7%) had a heterogeneous myometrium and 65.4% had concomitant presence of myoma. However, uterus length was ≥12 cm in 24.7% of cases. No other abnormalities were described in the clinical patient records and images or reports of these ultrasound scans were mostly unavailable, preventing any further evaluation.

**Figure 1:**
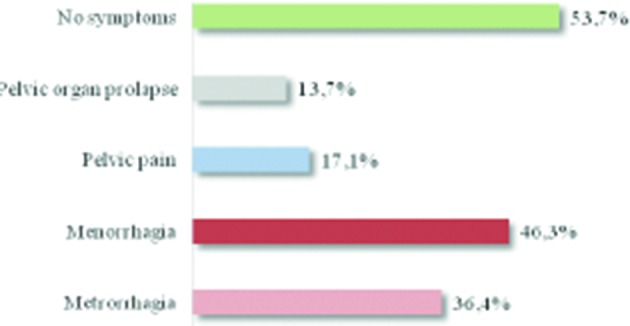
— Description of patient symptoms

**Figure 2:**
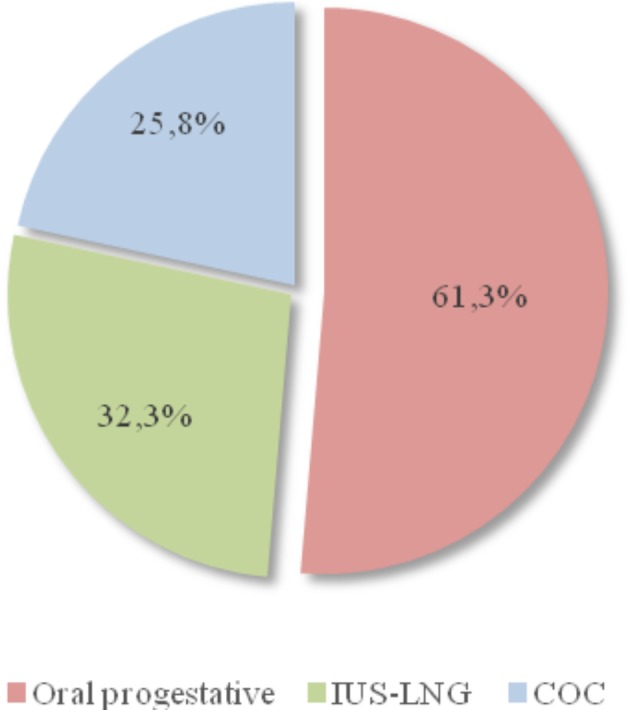
— Medical treatments used by women with symptoms. COC – combined oral contraceptive pills; IUS-LNG – intrauterine system with levonorgestrel.

More than a half of the women were asymptomatic (Fig. 1). When symptoms were present, menorrhagia and metrorrhagia were the most important complaints. However, pelvic pain and dysmenorrhea were recorded for 17.1% and 14.9% of women, respectively. Concerning surgical history, prior uterine surgery and previous caesarean section(s) were found in 26.3 % and 67.6% of cases respectively, while 17.7% mentioned a history of uterine curettage and 14.7% reported a myomectomy. Medical treatment with progestatives was used in the majority of women (Fig. 2) and 23.0% tried more than one type of hormonal therapy. None of them had been treated with a GnRH agonist.

Endometrial cancer was present in 5.5% of the women, which was well or moderately differentiated endometrioid adenocarcinoma in all cases.

## Discussion

Adenomyosis is a common benign gynaecological disorder affecting premenopausal women and can coexist with other uterine disorders, mainly with fibroids. In our study, the majority of women were asymptomatic (53.7%), which is a slightly higher proportion than that described in the literature (Taran et al., 2013; Sakhel et al., 2014; Graziano et al., 2015). However, symptoms are unspecific (abnormal uterine bleeding and chronic pelvic pain) and can simulate other diseases, such as endometriosis and endometrial carcinoma. Menometrorrhagia and increased menstrual flow were the main symptoms reported in this study (about 36 and 46% of women, respectively). However, uterine fibroids were present in 65.4%, therefore these symptoms could also be related to this finding.

Premenopausal age and multiparity are well-established risk factors that predispose to adenomyosis (Taran et al., 2013). In this study, more than 90% of women were aged over 40, multiparous, and 26.3% had had past uterine surgery, mostly caesarean section(s).

Adenomyosis may be difficult to diagnose based upon an ultrasound evaluation because different ultrasound features have been suggested to be linked with adenomyosis. At present, it is not clear which of the several ultrasound criteria are most important for diagnosis (Horng et al., 2014). In our study, heterogeneous myometrium was the only abnormality described and concomitant fibroids were present in more than 50% of women. However, the uterus length was more than 12 cm in less than one third. Because the definitive diagnosis of adenomyosis is based on a histological examination, usually of a hysterectomy sample, adenomyosis is a neglected diagnosis. Certain ultrasound features might prove to be more clinically relevant than others, but more research on this is necessary.

The goal of medical therapy is the improvement of symptoms. The results of systematic literature reviews have consistently demonstrated that as long as amenorrhea is achieved, there are no statistical differences between the various available drugs in terms of pain relief, but tolerability, side effects, and cost vary widely (Tsui et al., 2014). Medical management can be effective, as with the management of myoma, and this is the first therapeutic option for women who want to conceive. However, its effect is often transient and it is frequently used with a preoperative adjuvant therapy, or sometimes a postoperative therapy (Horng et al., 2014). Rapid regrowth of adenomyosis and relapse of symptoms and signs always occurs once the treatment is discontinued. Therefore, other alternative strategies might be needed. The effectiveness of conservative uterine surgery is promising. Although, women have to be clearly informed about negative outcomes on fertility. A recent review showed that the reproductive performance rate of women who underwent combined surgical and medical treatment for adenomyoma was 41.1%, and the majority of pregnancies occurred within the first year after treatment, suggesting that these women’s ability to conceive was reduced by 25-33% each year after the completion of therapy (Chang et al., 2013). This effect on reproductive performance in women with diffuse-type adenomyosis is less promising. In these cases, the pregnancy rate was 30-40% (Wang et al., 2009). Additionally, because of the recent trend of delayed marriage and childbearing, premenopausal adenomyosis might increasingly become a factor affecting fertility, which makes the presence of adenomyosis more frequent. It is therefore important to carry out more research and invest in conservative therapeutic options. Hysterectomy remains the most important therapeutic option for women with symptomatic adenomyosis who have completed their reproductive project (Horng et al., 2014; Shu et al., 2016).

Adenocarcinoma arising from adenomyosis is rare. There are two pathways of carcinogenesis for this condition: a) *de novo* malignant transformation of adenomyotic foci while the eutopic endometrium was unaffected; b) simultaneous malignant changes in the eutopic endometrium and adenomyosis. When an endometrial carcinoma and adenomyosis coexist, adenomyosis is invaded by the carcinoma in approximately 25% of cases. An association between adenomyosis and other oestrogen-dependent benign diseases such as endometrial polyps is common, suggesting that hyperoestrogenic state may share the pathogenesis of these gynaecological diseases and endometrial cancer. Endometrial cancers involving adenomyosis have been found to be associated with a low histologic grade, a history of hormone use and more favourable prognosis (Verit and Yucel, 2013).

In our study, 5.5% of cases had low-grade endometrioid adenocarcinoma, similar to the figure that can be found in the literature. Four cases of carcinoma (0.74 %) were reported in 564 patients operated on between 1981 and 2001 (Koshiyama et al., 2002). Out of 219 patients with the diagnosis of early endometrial cancer, malignant changes in adenomyosis were present in 6.8% (Kucera et al., 2011). Nevertheless, diagnosis is often delayed because of the absence of any lesion in the eutopic endometrium.

Missing clinical information (clinical patient records occasionally incomplete, and lack of access to the ultrasound images and full reports), as well as the retrospective approach are the most important limitations of this study. Strong points are that all cases were selected based on histological evidence and that few articles about adenomyosis have been published, most of which are theoretical reviews. More studies and case-control approaches are needed to improve the diagnosis and management of this complex disease.

## Conclusion

Adenomyosis is an important clinical challenge in gynaecology. Women with adenomyosis are often asymptomatic. Symptoms are unspecific and can be severe, leading to a decrease in quality of life indexes, but the symptoms can also be a manifestation of other concomitant disorders such as uterine fibroids.

To allow an early diagnosis, clinicians should look carefully for ultrasound features associated with adenomyosis. To confirm the diagnosis MRI can be required.

Symptomatic women receiving treatment for adenomyosis are mostly in their fourth or fifth decade of life and multiparous. The therapeutic goal of medical treatment is directed towards symptom control and the wish to conceive. Surgical treatment is the most effective treatment in terms of clinical improvement in symptomatic adenomyosis. The choice for a surgical approach is dependent on the women´s wish to preserve fertility. Adenocarcinoma arising from adenomyosis is rare and is commonly a low-grade endometrioid adenocarcinoma.

Adenomyosis often remains an unexpected post-operative finding after hysterectomy. Future investments in the establishment of diagnostic algorithms and supplementary treatments are warranted.

**Table II:** — Histopathological results of hysterectomy specimens.

	**Minimum**	**Maximum**	**Mean**
Uterus weight	31 g	1544 g	235.6 ± 232.7
Uterus length	6 cm	17 cm	10.0 ± 2.3
Myometrium thickness	10 mm	60 mm	23.6 ± 0.7
